# Patient-reported outcomes of a randomized phase III clinical trial of adjuvant radiation versus chemoradiation in intermediate risk, stage I/IIA cervical cancer patients treated with initial radical hysterectomy and pelvic lymphadenectomy (NRG/GOG-0263)

**DOI:** 10.1016/j.ygyno.2025.09.014

**Published:** 2025-10-06

**Authors:** Dana M. Chase, Helen Q. Huang, Wei Deng, Wui-Jin Koh, William Rodgers, William Small, Kevin Albuquerque, Jyoti Mayadev, Charles A. Leath, Bradley Monk, Beob-Jong Kim, Dae-Yeon Kim, Chi Heum Cho, Jae-Weon Kim, Jae Hong No, Laura Holman, Ashley Stuckey, Denise Fabian, Alexandra H. Smick, Lari Wenzel, Karen Gil, Sang Young Ryu

**Affiliations:** a Division of Gynecologic Oncology, David Geffen School of Medicine UCLA, Los Angeles, CA, USA; b NRG Oncology Statistics and Data Management Center, Roswell Park Comprehensive Cancer Center, Buffalo, NY, USA; c National Comprehensive Cancer Network, Plymouth Meeting, PA, USA; d Department of Radiation Oncology, Weill Cornell Medicine, New York, NY, USA; e Department of Radiation Oncology, Stritch School of Medicine, Cardinal Bernadin Cancer Center, Loyola University Chicago, Maywood, IL, USA; f Department of Radiation Oncology, UT Southwestern Medical Center, Dallas, TX, USA; g Department of Radiation Medicine and Applied Sciences, University of California San Diego, La Jolla, CA, USA; h Division of Gynecologic Oncology, University of Alabama at Birmingham, Birmingham, AL, USA; i Department of Obstetrics and Gynecology, Creighton University School of Medicine at St. Joseph’s Hospital and Medical Center, Phoenix, AZ, USA; j Department of Gynecologic Oncology, Korean Cancer Center Hospital, Seoul, South Korea; k Department of Obstetrics and Gynecology, Asan Medical Center, Seoul, South Korea; l Department of Obstetrics and Gynecology, Keimyung University Dongsan Medical Center, Daegu, South Korea; m Department of Obstetrics and Gynecology, Seoul National University Hospital, Seoul, South Korea; n Department of Obstetrics and Gynecology, Seoul National University Bundang Hospital, Seongnam, South Korea; o Division of Gynecologic Oncology, University of Oklahoma Health Sciences Center, Oklahoma City, OK, USA; p Department of Obstetrics and Gynecology, Women and Infants Hospital, Providence, RI, USA; q Department of Radiation Oncology, University of Kentucky, Lexington, KY, USA; r Department of Population Health and Disease Prevention, University of California, Irvine, CA, USA; s Department of Obstetrics and Gynecology, Northeast Ohio Medical University, Rootstown, OH, USA; t Department of Radiation Oncology, Korea Institute of Radiological and Medical Sciences, Seoul, South Korea

**Keywords:** Cervical cancer, Chemoradiotherapy, Adjuvant therapy, Quality of life, Patient-reported outcomes

## Abstract

**Objective.:**

To prospectively evaluate the impact of adjuvant chemoradiation (RT + CIS) versus radiation (RT) on quality of life (QOL) and patient-reported outcomes (PROs) among patients with intermediate-risk, stage I-IIA cervical cancer treated with radical hysterectomy and pelvic lymphadenectomy.

**Methods.:**

Patients enrolled in GOG-0263 completed PRO/QOL assessments at baseline, 3, 7, and 36 weeks using the FACT-Cx Trial Outcome Index (FACT-Cx TOI), FACT/GOG-Neurotoxicity subscale (FACT/GOG-Ntx-4), the worst pain item from the Brief Pain Inventory (BPI), and five gastrointestinal/genitourinary (GI/GU) symptom items. Linear mixed models adjusted for baseline score, treatment, age, performance status, and country.

**Results.:**

Among 316 randomized eligible patients (RT + CIS: *n* = 158; RT: n = 158), questionnaire completion rates were 98 %, 90 %, 88 %, and 81 % at baseline, weeks 3, 7, and 36, respectively. Patients receiving RT + CIS reported a mean FACT-Cx TOI score 5.1 points lower than RT at 3 weeks (97.5 % CI: −8.6 to −1.6; *p* = 0.004) and 6.3 points lower at 7 weeks (97.5 % CI: −10.2 to −2.4; *p* = 0.002). By 36 weeks, scores had returned to baseline in both groups, with no significant difference (*p* = 0.386). Patient-reported neuropathy scores (FACT/GOG-Ntx-4) did not differ significantly between groups at any time point (*p* = 0.82). Patient-reported GI/GU symptoms and pain worsened at 3 weeks in both arms, followed by recovery to baseline by 36 weeks.

**Conclusion.:**

QOL declined in both groups after treatment initiation, with greater short-term deterioration in the RT + CIS group. By 36 weeks, QOL and other PROs returned to baseline in both groups. Neuropathy, GI/GU symptoms, and pain showed no significant differences between treatment arms over time.

## 1. Introduction

The integration of patient-reported outcomes, or PROs, into clinical trials, has evolved over the past few decades. Early cervical cancer clinical trials, like GOG 92 (radiation therapy vs observation after surgery) and 109 (chemoradiation versus radiation after surgery), focused primarily on objective endpoints such as progression-free survival (PFS) and overall survival (OS) [1,2]. However, researchers and regulatory agencies recognized that these measures alone did not capture the full impact of treatments on patients’ daily lives. The Food and Drug Administration (FDA) began encouraging the inclusion of patient reported outcomes (PROs) in clinical trials in the early 2000s, leading to the development of standardized tools to assess quality of life (QOL), symptom burden, and functional status [3].

After the design and implementation of GOG-0263, several cervical cancer clinical trials have reported on PROs. These studies have either advocated for a novel therapy and/or given insight into patient-reported adverse events of therapy. For example, in one recurrent metastatic cervical cancer trials, QOL improvements were seen with checkpoint inhibitor therapy, more so than with traditional chemotherapy [4]. In another trial, the addition of checkpoint inhibitor therapy to standard of care chemotherapy did not worsen QOL/PROs and improvements in QOL as well as less deterioration in QOL were seen in the experimental arm [5]. In locally advanced cervical cancer trials, many QOL measures either improved or were similar with novel therapies; however, sexual function, neuropathy and menopausal symptoms worsened and thus provided insight in the patient experience [6,7]. Less aggressive and/or traditional laparotomy surgical approaches for early cervical cancer patients were associated with improved QOL/PROs, helping to advocate for surgical approach based on QOL outcomes [8,9]. Hence, clinical trial data that incorporated PROs have shed more insight into novel therapies than traditional trial outcomes.

GOG-0263 was a study of the addition of cisplatin to radiation therapy for stage I/IIA intermediate risk cervical cancer patients after radical hysterectomy and pelvic lymphadenectomy to determine if cisplatin improves recurrence free survival (RFS). It was the first US-based clinical trial to measure prospective PROs in early-stage cervical cancer patients. From prior studies, it was known that the addition of cisplatin to radiation resulted in increased rates of clinician assessed toxicities [2]. For GOG 263, there was interest in whether PROs measures similarly reflect these increased rates of adverse events.

In general, PROs are measured much less frequently than the collection of adverse events and even a single episode of a high-grade toxicity will be included in the results of the study. PROs are not collected daily, weekly or even at every patient visit. In GOG-0263, grade 3/4 hematologic (especially neutropenia) and gastrointestinal (GI) toxicities were higher with the addition of chemotherapy to radiation [10]. There were also higher rates of grade 1/2 neuropathy with the addition of cisplatin compared to radiation alone. Thus, the hypothesis was that patients on GOG-0263 would reflect worsening PROs when cisplatin was added to radiation therapy.

## 2. Methods

### 2.1. Enrollment

GOG-0263 (NCT01101451) was a randomized phase 3 trial conducted in the United States, Korea, and Japan (https://www.clinicaltrials.gov/search?term=NCT%2001101451). Patients were eligible for this study if they had FIGO 2009 stage I-IIA cervical cancer with squamous cell carcinoma, adenocarcinoma, or adenosquamous carcinoma histology and were treated with standard radical hysterectomy with pelvic lymphadenectomy (open or laparoscopic). Additional eligibility criteria required high-intermediate risk features on final surgical pathology. Patients with positive lymphovascular space invasion (LVSI) were eligible if they had one of the following: deep third stromal invasion, middle third stromal invasion with a clinical tumor ≥2 cm, or superficial third stromal invasion with a clinical tumor ≥5 cm). Patients with negative LVSI were eligible if they had middle or deep third stromal invasion with a clinical tumor ≥4 cm (Table 1). A GOG performance status of 0 to 2 was required, and patients had to have adequate renal, hepatic, and bone marrow function. Exclusion criteria included patient less than 18 years old, evidence of severe infection, intestinal obstruction or gastrointestinal bleeding, postoperative fistula, prior chemotherapy or radiation for cervical cancer, anticipated modification of the radiation field, or a history of invasive malignancy (except non-melanoma skin cancer) within the past five years.

### 2.2. Study design and treatment

Patients meeting eligibility criteria were randomized 1:1 after surgery to either adjuvant radiotherapy (RT) alone or concurrent chemoradiotherapy with weekly cisplatin (RT + CIS). Randomization was conducted using a dynamic allocation (minimization) algorithm to ensure balance across the following stratification factors: LVSI (positive vs. negative), depth of stromal invasion (deep, middle, superficial), performance status (0–1 vs. 2), external beam radiotherapy (EBRT) modality (standard 4-field vs. Intensity Modulated Radiation Therapy (IMRT), and cooperative group (GOG vs. KGOG vs. other).

All patients were scheduled to receive EBRT, initiated 3–8 weeks postoperatively. Treatment was delivered either as conventional 4-field radiotherapy or IMRT, to a total dose of 50.4 Gy in 28 fractions (1.8 Gy/fraction), once daily, five days per week, over approximately 5.5 weeks. For IMRT, credentialing by Imaging and Radiation Oncology Core (IROC) Houston was required, and the volume specifications are described RTOG Gynecological Atlas on website: http://www.rtog.org/atlases/gynatlas/main.html. Brachytherapy boost was not allowed on this protocol.

Patients in the RT + CIS arm received up to 6 cycles of concurrent weekly cisplatin at a dose of 40 mg/m2, preferably administered on Mondays and approximately four hours before RT. The 6th cycle of cisplatin could be omitted if all RT was completed. Details on chemotherapy dose modifications and adverse event management are provided in the trial protocol.

### 2.3. Quality of life assessments

The FACT-Cx (Functional Assessment of Cancer Therapy-Cervix) is a validated, self-report instrument composed of the FACT-G (General) questionnaire with an additional cervical cancer-specific subscale, totaling 42 items [11]. It includes five subscales: physical well-being (PWB), functional well-being (FWB), social/family well-being (SWB), emotional well-being (EWB), and the cervical cancer subscale (CCS). All items are rated on a 5-point Likert scale (0 = not at all to 4 = very much), with higher scores indicating better QOL. For the negative statements (or questions), reversal was performed prior to score calculation. The total FACT-Cx score ranges from 0 to 168. For this trial, the FACT-Cx Trial Outcome Index (Fact-Cx TOI) was used to assess overall QOL in a clinical trial context, with a score range of 0 to 116. This is a validated subset composed of the PWB, FWB, and CCS subscales.

The FACT/GOG-Ntx-4 is a validated 4-item neurotoxicity subscale used to assess patient-reported symptoms of peripheral neuropathy associated with chemotherapy [12]. Each item is scored on the same 0–4 Likert scale, with a total possible score of 0 to 16, and higher scores indicating less neurotoxicity. This abbreviated version was selected to capture neurotoxicity while minimizing respondent burden.

Five single-item PROs were included to capture gastrointestinal (GI) and genitourinary (GU) symptoms: “I urinate more frequently than usual,” “I have control of my bowels,” “I have cramps in my stomach area,” “I can digest my food well,” and “I have diarrhea.” The scoring of these items followed the same 5-point Likert scale (0 = not at all to 4 = very much) so that a higher score reflected fewer or less severe symptoms. These additional items were included to enhance sensitivity to mild acute treatment-related toxicity not captured by the standard adverse event grading systems.

One item from the Brief Pain Inventory (BPI) was included to assess the worst pain experienced in the past 24 h. This item is scored from 0 (no pain) to 10 (worst imaginable pain), with higher scores indicating greater pain severity [13].

The above questionnaires were translated into Korean and validated by the Center on Outcomes, Research and Education (CORE) at Evanston Northwestern Healthcare in Evanston, IL. In Japan, the forms were completed mostly in English (*N* = 13), with a 4 completing the forms in Korean. This translation process followed a rigorous methodology that included forward translation, back translation, cognitive interview, and psychometric testing to ensure conceptual equivalence and cross-cultural validity. This approach incorporated the expertise of a multidisciplinary translation team and staff experienced in health outcomes measurements using previously published methodology [14–16]. Additional validation studies have confirmed the reliability and cultural appropriateness of the Korean version of the BPI, including back translation and psychometric evaluation in Korean patient populations [17].

### 2.4. Data collection

The QOL/PRO Questionnaires were administered in-person, using paper-based Scantron forms and were distributed by either a physician, nurse, or data manager at four separate time points: baseline (prior to randomization), and at 3, 7 and 36 weeks after initiation of study treatment. For patients receiving concurrent cisplatin, the questionnaire was completed prior to receiving the chemotherapy infusion on the day of assessment. Patients were permitted to request assistance with the questionnaire, but family members were instructed to wait outside the room during questionnaire completion to reduce potential bias. The questionnaires were available in both English and Korean to accommodate participating institutions.

### 2.5. Outcomes

The primary clinical objective of GOG-0263 was to determine whether postoperative chemoradiotherapy (CIS + RT) improves RFS compared to radiation therapy (RT) alone in FIGO 2009 stage I-IIA cervical cancer patients with intermediate-risk pathologic features following radical hysterectomy. The secondary clinical object was to assess whether CIS + RT improves OS compared to RT alone. The outcomes of these clinical endpoints are reported separately [10].

The primary QOL and PROs objectives were to evaluate the impact of CIS + RT on health-related QOL compared to RT alone, as measured by the FACT-Cx TOI, and on chemotherapy-related neurotoxicity, as assessed by the FACT-GOG-NTx-4. Exploratory analyses included five additional patient-reported GI and GU symptoms, and pain severity using the BPI worst pain score.

### 2.6. Statistical analysis

The QOL objectives were secondary and designed to compare health-related QOL, measured with the FACT-Cx TOI, and patient-reported neurotoxicity, as measured with the FACT/GOG-Ntx-4 subscale, between the two treatment groups. To control the overall type I error at 5 %, a Bonferroni-adjusted significance level of 0.025 (0.05/2) was applied to the two co-primary QOL and PRO endpoints. The QOL and PROs hypotheses were not included in the sample size calculation, as this was based on the primary clinical endpoint of RFS. All analyses were undertaken using SAS/STAT Software 9.4.

For the FACT-Cx and FACT/GOG-Ntx-4, negatively worded items were reverse-coded prior to scoring. Subscale scores were calculated as the sum of individual item scores when more than 50 % of items in the subscale were completed. For partially missing data, prorated scores were computed by multiplying the mean of the completed items by the total number of items in the subscale.

Treatment differences in QOL and PROs were assessed using linear mixed models adjusted for baseline score, treatment assignment, age, performance status at enrollment, and country of treatment. Analyses were conducted based on the intention-to-treat principle, with patients classified according to their randomized assignment. Assessment time points were treated as categorical variables due to unequal spacing. An unstructured covariance matrix was used to account for the correlation of repeated measures within subjects, and empirical (sandwich) standard errors were used to estimate the precision of model parameters. Interaction terms between treatment assignment and time were tested at a significance level of 0.05 to assess whether treatment effects varied over time. If no significant interaction was detected, an overall treatment effect was estimated as a weighted average across all time points. If the interaction was statistically significant, treatment effects were estimated separately at each time point, with *p*-values adjusted using Hochberg’s step-up procedure [18]. Treatment differences were reported with 97.5 % confidence intervals for the primary QOL and PROs outcomes. Comparisons of the five additional symptoms and the BPI worst pain score were conducted as exploratory analyses using similar methods, with 95 % confidence intervals reported.

PRO compliance by the two regimens was compared with the generalized estimating equation (GEE) method.

## 3. Results

### 3.1. Patients

**Fig. 1.**
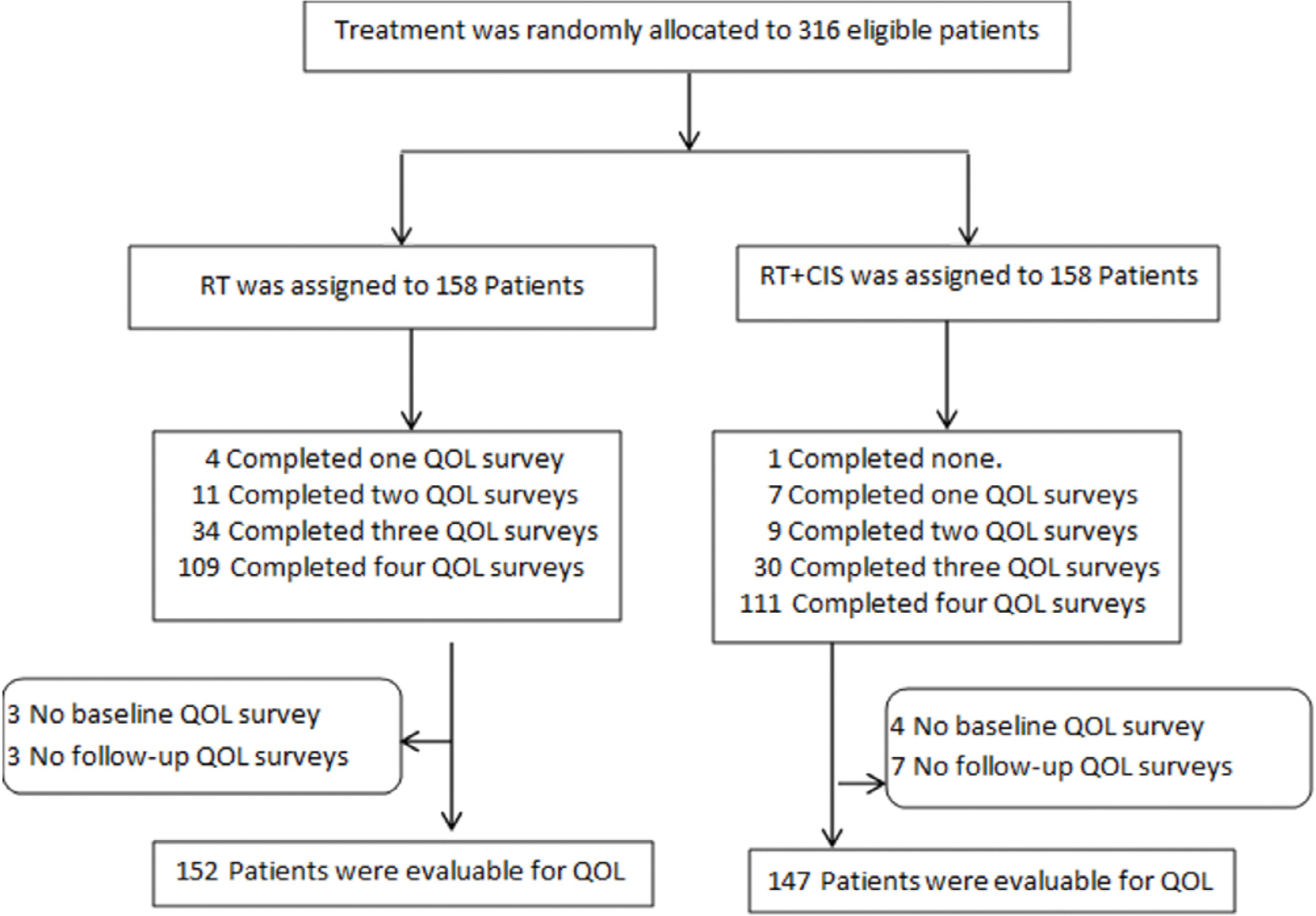
Consort diagram of patients eligible for PRO/QOL analysis.

Between April 12, 2010, and April 11, 2022, 340 patients were enrolled to GOG-0263 and randomized including 316 eligible (RT; *n* = 158, or RT + CIS; n = 158). Patients were considered evaluable for PRO/QOL endpoints if they completed the baseline questionnaire and at least one follow-up assessment. Based on these criteria, 152 patients in the RT group and 147 patients in the RT + CIS group were included in the PRO/QOL analysis (Fig. 1).

PRO/QOL questionnaire compliance was high across all time points. Overall, 98 % of patients completed the baseline questionnaire, 90 % completed the 3-week questionnaire, 88 % completed the 7-week questionnaire, and 81 % completed the 36-week questionnaire (Table 1). Reasons for missing assessments included severe illness or treatment-related toxicities precluding reporting, institutional error, patient refusal, lost to follow-up, insufficient answers, and withdrawal (Table 1). Compliance rates did not differ significantly between the RT and RT + CIS groups at any time point (*p* = 0.7).

Among the 299 patients included in the PRO/QOL analysis, the mean age was 48 years. The total cohort was 53 % Asian and 38 % White, and 85 % had an ECOG performance status of 0. LVSI was present in 74 % of patients and 61 % had deep third stromal invasion. There were no significant differences in baseline patient or tumor characteristics between the RT and RT + CIS groups (Table 2).

### 3.2. FACT-Cx TOI score

**Fig. 2.**
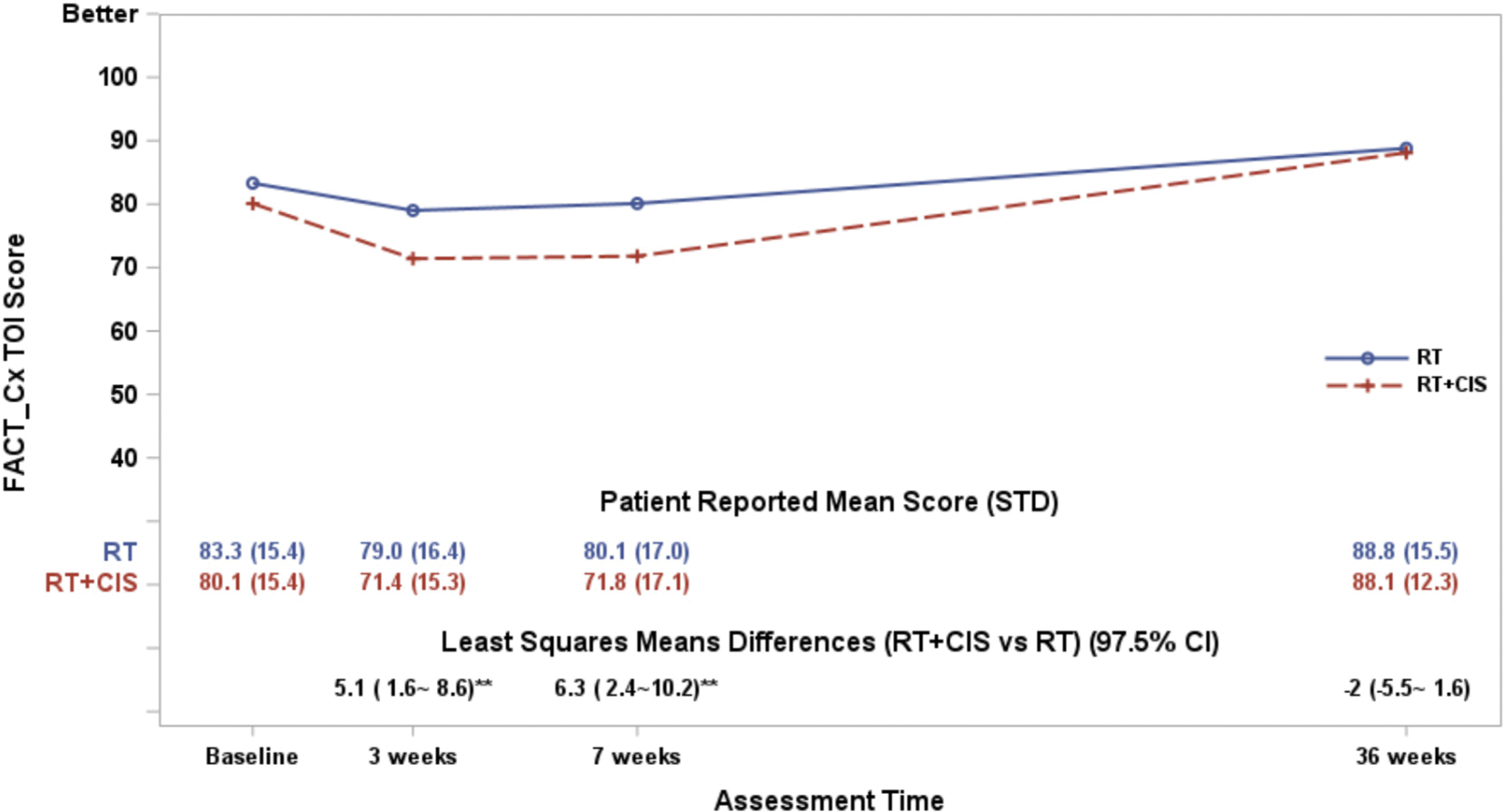
The plot lines represent the FACT-Cx TOI scores. The estimated least-squares means (LSM) differences were obtained from a fitted mixed model adjusting for pre-treatment score (baseline score), patient’s age and performance status at the enrollment, and the country the treatment was administered. A larger score indicates favorable or better QOL. Note “**” indicates Hochberg adjusted p-value <0.01.

At baseline, the mean FACT-Cx TOI score was 83.3 in the RT group and 80.1 in the RT + CIS group. Both groups experienced a decline in QOL at 3 weeks following treatment initiation, with a greater reduction observed in the RT + CIS group (Fig. 2). QOL gradually improved over time in both groups, with scores exceeding baseline levels by 36 weeks. After adjusting for age, ECOG performance status, baseline score, and treatment country, a linear model revealed interaction between the treatment and assessment time (*p* < 0.0001) with significantly lower QOL in the RT + CIS group compared to RT at both 3 and 7 weeks. At 3 weeks, patients receiving RT + CIS reported a mean score 5.1 points lower than those receiving RT (97.5 % CI: −8.6 to-1.6; Hochberg adjusted *p* = 0.004), and at 7 weeks, the difference was 6.3 points (97.5 % CI: −10.2 to −2.4; Hochberg adjusted *p* = 0.002). By 36 weeks, there was no significant difference in QOL scores between treatment groups (Hochberg adjusted *p* = 0.386).

There were 144 US patients, 138 Korean patients and 17 Japanese patients in this study who were evaluable for QOL (Table S1). At baseline, Korean patients reported 8.2 points lower QOL (95 % CI: 4.5–11.9) than US patients and 8.1 points lower QOL (95 % CI: 0.3–15.9) than Japanese patients in the FACT-Cx TOI score. The differences between countries were consistent in each treatment group (no interaction effects between treatment groups and countries; *p* = 0.92). After adjustment for treatment groups, age, performance status, assessment time, and the baseline scores, the Korean patients reported 1.4 points lower QOL (95 % CI: −1.4–4.1) than US patients on average over follow-up time points. In general, the Korean patients reported a lower FACT-Cx TOI score than those in Japan or the US, but these location differences existed across the treatment groups. Therefore, the country effect was not significantly associated with the treatment differences in the FACT-Cx TOI score.

### 3.3. FACT/GOG-Ntx-4 score

**Fig. 3.**
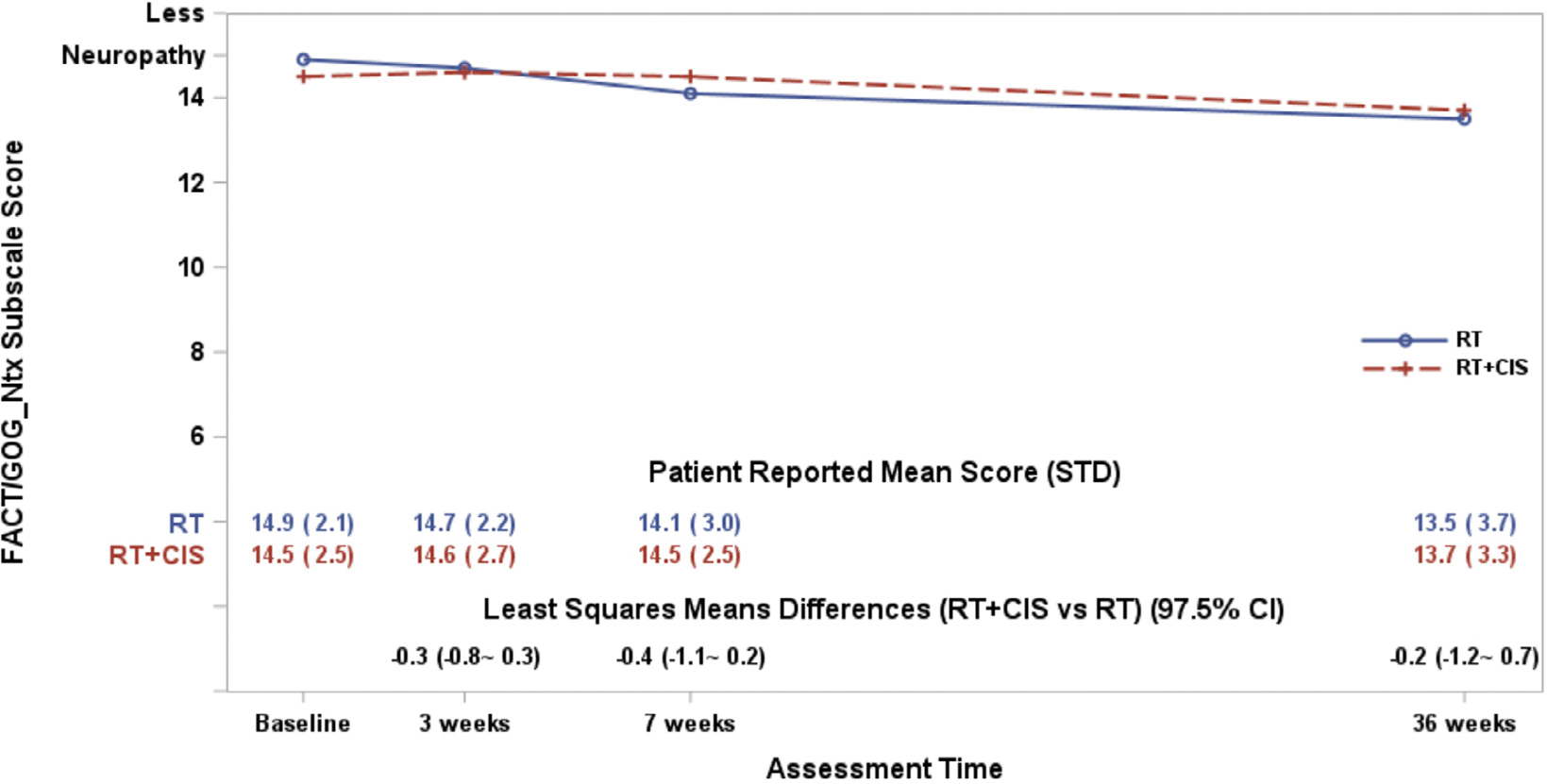
The plot lines represent the FACT/GOG-Ntx-4 scores. The estimated least-squares means (LSM) differences were obtained from a fitted mixed model adjusting for pre-treatment score (baseline score), patient’s age and performance status at the enrollment, and the country the treatment was administered. A larger score indicates less neurotoxic symptoms.

At baseline, the mean score was 14.9 in the RT group and 14.5 in the RT + CIS group. After adjusting for age, ECOG performance status, baseline score, and treatment country, there were no statistically significant differences in patient-reported neuropathy between treatment groups at any time point (interaction *p* = 0.82) (Fig. 3). The estimated mean difference over time was 0.32 points higher in the RT + CIS group compared to the RT group, which was not statistically significant (97.5 % CI: −0.14 to 0.79; *p* = 0.12).

### 3.4. Patient-reported symptoms

**Fig. 4.**
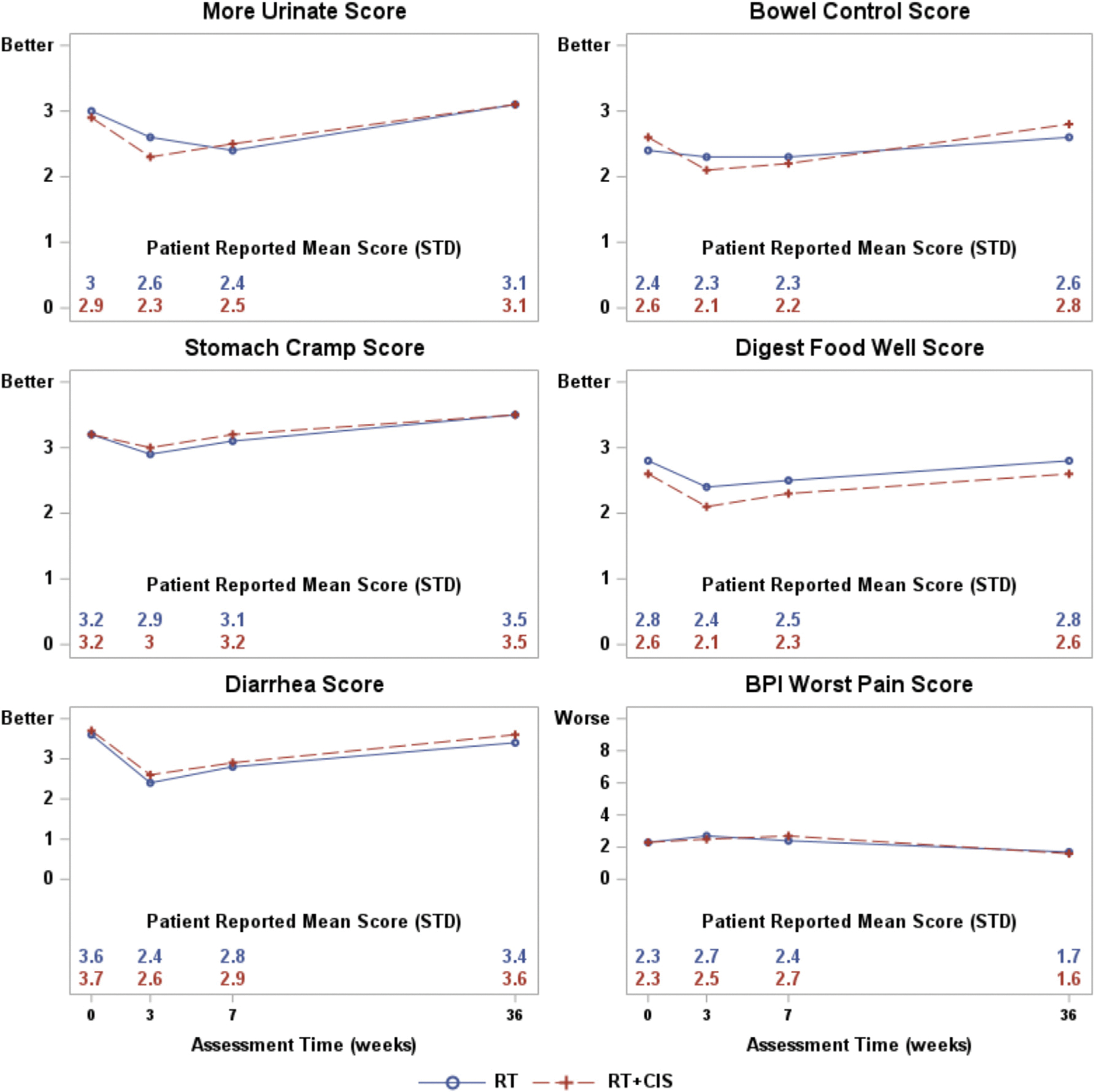
The plot lines represent the patient-reported symptoms and pain scores. A larger score indicates better or favorable status except for the BPI worst pain score in which a larger score indicates worse pain.

For all items, symptoms worsened at 3 weeks in both treatment groups, followed by gradual improvement to baseline by 36 weeks (Fig. 4). After adjusting for age, ECOG performance status, baseline score, and treatment country, there were no statistically significant differences in these patient-reported symptoms between the RT and RT + CIS groups at any time point (Fig. 4). Both groups reported an increase in pain at 3 weeks, with resolution and improvement beyond baseline levels by 36 weeks. Adjusted analyses also showed no statistically significant differences in BPI pain scores between treatment groups at any time point.

## 4. Discussion

In this trial of adjuvant therapy for intermediate-risk cervical cancer, the addition of chemotherapy to radiation did not statistically improve clinical outcomes including both RFS and OS as hypothesized. Although this was a negative trial for both RFS and OS, it contributes meaningfully to our understanding of the patient experience with adjuvant therapy in early-stage cervical cancer. A decline in health-related QOL was observed in both treatment arms approximately three weeks after starting therapy, with significantly greater deterioration in the RT + CIS group at both 3 and 7 weeks. By nine months, QOL had returned to baseline in both groups, although completion rates for QOL/PRO questionnaires fell below 90 % at that time. There may be a numerical improvement at nine months compared to baseline which may reflect further recovery from primary surgical efforts.

This is the first prospective randomized study in this patient population to report QOL outcomes. No other trials in intermediate- or high-risk early-stage cervical cancer have reported longitudinal PROs [19]. In contrast, PROs have been more commonly included in trials of locally advanced cervical cancer, where patients receive definitive chemoradiation without surgery [6,7,20]. These studies, evaluating either the addition of checkpoint inhibitors or the use of induction chemotherapy before chemoradiation, have reported grade 3/4 toxicities in 60–70 % of patients and all-grade diarrhea in approximately 50 % of patients in both arms. At 36 weeks, around 60 % of patients completed all QOL questionnaire items, while over 95 % completed at least one item in locally advanced cervical cancer studies. In one trial evaluating chemoradiation with or without induction chemotherapy, both groups experienced notable declines in QOL during weeks 3–4 of treatment, primarily due to diarrhea, nausea/vomiting, and fatigue, with symptom resolution by week 12 [7]. Similarly, GOG-0263 illustrated declines in QOL during the active treatment period (i.e., weeks 3 and 7), with significantly worse QOL in the RT + Cis treatment arm. Taken together, and consistent with our findings, results highlight the need for enhanced supportive care especially during adjuvant treatment in patients receiving radiation with or without chemotherapy.

This study has several limitations. First, while patient-reported GI and GU symptoms were similar between the two treatment groups, GOG-0263 was not a placebo-controlled trial. As a result, patients receiving RT + CIS may have had more frequent office or infusion center visits, potentially increasing opportunities for supportive care interventions. Second, although cisplatin is known to increase treatment-related toxicity, QOL declined in both groups between weeks 3 and 7, regardless of treatment assignment. This highlights the challenge of comparing clinician assessed toxicities with patient-reported outcomes. Additionally, toxicities and PROs were collected on different timelines, and clinical measures such as laboratory abnormalities may not fully reflect how patients perceive or experience adverse effects. Finally, there were differences in QOL reporting by country, with Korean patients reporting lower QOL scores on average compared to those from the United States or Japan. Whether these differences reflect cultural differences, language translation, or clinical practice patterns remains unclear. These limitations underscore the importance of incorporating PROs into clinical trials to better capture patient experience and inform supportive care strategies.

These observations highlight the ongoing need to develop interventions that meaningfully address the patient experience during adjuvant treatment for early-stage cervical cancer. Few strategies have been specifically tested to reduce treatment-related toxicity in this setting, particularly during weeks 3 to 4 of treatment. It is worth noting that many clinical trials now assess patient-reported ‘bother with side effects of treatment’, using the GP5 single-item from the FACT Physical Well-Being subscale. This specific item may inform strategies to reduce toxicities. Some trials have explored approaches such as transdermal versus oral antiemetics, probiotics, dietary modifications, therapeutic interventions, or antioxidant enemas to alleviate nausea, proctitis, and diarrhea [21–23]. Future research should prioritize supportive care strategies both during treatment and throughout the post-treatment recovery period. Continued integration of PROs into cervical cancer clinical trials will be essential to inform therapeutic development, optimize patient-centered care, and improve survivorship outcomes.

## Figures and Tables

**Table 1:** PRO/QOL questionnaire compliance rates by time point.

Time points	Compliance Status	RT (*n* = 158)	RT + CIS (*n* = 158)	Total (*n* = 316)

	Complete, n (%)	155 (98 %)	154 (97 %)	309 (98 %)
	Missed due to:			
Baseline	Illness/toxicities	1	0	1
	Institutional error	1	2	3
	Lost to follow-up	0	1	1
	Insufficient answer	1	1	2
	Complete, n (%)	140 (89 %)	143 (91 %)	283 (90 %)
	Missed due to:			
	Illness/toxicities	0	1	1
	Refusal	6	2	8
3 weeks	Institutional error	9	4	13
	Lost to follow-up	0	4	4
	Other	3	1	4
	Insufficient answer	0	1	1
	Withdrawal^b^	0	2	2
	Complete, n (%)	143 (91 %)	134 (85 %)	277 (88 %)
	Missed due to:			
	Illness/toxicities	0	1	1
	Refusal	2	3	5
7 weeks	Institutional error	6	8	14
	Lost to follow-up	0	7	7
	Other	6	3	9
	Insufficient answer	1	0	1
	Withdrawal^b^	0	2	2
	Complete, n (%)	126 (80 %)	128 (81 %)	254 (81 %)
	Missed due to:			
	Illness/toxicities	0	2	2
	Refusal	4	2	6
36 weeks	Institutional error	17	5	22
	Lost to follow-up	5	8	13
	Other	5	7	12
	Insufficient answer	0	1	1
	Withdrawal^b^	2	1	3

b Number of withdrawals is cumulative up to each time points.

**Table 2:** Characteristic of patients included in PRO/QOL analysis.

Patient Characteristics	RT (*n* = 152)	RT + CIS (*n* = 147)	Total (*n* = 299)

Age group, n (%)			
≤39	10 (6.6)	19 (12.9)	29 (9.7)
40–49	58 (38.2)	48 (32.7)	106 (35.5)
50–59	45 (29.6)	42 (28.6)	87 (29.1)
60–69	30 (19.7)	25 (17.0)	55 (18.4)
≥70	9 (5.9)	13 (8.8)	22 (7.4)
Race, n (%)			
White	59 (38.3)	55 (37.4)	114 (38.1)
Asian	77 (50.7)	81 (55.1)	158 (52.8)
Black/African American	11 (7.2)	3 (2.0)	14 (4.7)
Other	5 (3.3)	8 (5.4)	13 (4.3)
Ethnicity, n (%)			
Hispanic	12 (7.9)	15 (10.2)	27 (9.0)
Non-Hispanic	137 (90.1)	131 (89.1)	268 (89.6)
Other/Unspecified	3 (2.0)	1 (0.7)	4 (1.3)
ECOG performance status, n (%)			
0	133 (87.5)	121 (82.3)	254 (84.9)
1	19 (12.5)	24 (16.3)	43 (14.4)
2	0 (0)	2 (1.4)	2 (0.7)
Country, n (%)			
Japan	6 (3.9)	11 (7.5)	17 (5.7)
South Korea	70 (46.1)	68 (46.3)	138 (46.2)
United States	76 (50.0)	68 (46.3)	144 (48.2)
Stage at diagnosis, n (%)			
I	142 (93.4)	136 (92.5)	278 (93.0)
II	10 (6.6)	11 (7.5)	21 (7.0)
LVSI, n (%)			
Negative	39 (25.7)	38 (25.9)	77 (25.8)
Positive	113 (74.3)	109 (74.1)	222 (74.2)
Stromal invasion, n (%)			
Deep	87 (57.2)	94 (63.9)	181 (60.5)
Middle	58 (38.2)	51 (34.7)	109 (36.5)
Superficial	7 (4.6)	2 (1.4)	9 (3.0)

ECOG = Eastern Cooperative Oncology Group; LVSI = lymphovascular space invasion.

## HIGHLIGHTS

- Health-related QOL declines significantly at 3 and 7 weeks during intermediate risk early cervical cancer treatment.
- When cisplatin is added to radiation, patients with intermediate risk cervical cancer have steeper declines in QOL.
- Because clinician-assessed toxicities and PROs are collected on different timelines, they are difficult to compare.
- Future research should prioritize supportive care strategies during treatment and in the post-treatment recovery period.
